# A combination of YM-155, a small molecule survivin inhibitor, and IL-2 potently suppresses renal cell carcinoma in murine model

**DOI:** 10.18632/oncotarget.4121

**Published:** 2015-05-12

**Authors:** Kai Guo, Peng Huang, Naijin Xu, Peng Xu, Haruki Kaku, Shaobo Zheng, Abai Xu, Eiji Matsuura, Chunxiao Liu, Hiromi Kumon

**Affiliations:** ^1^ Department of Urology, Zhujiang Hospital, Southern Medical University, Guangzhou, People's Republic of China; ^2^ Department of Urology, Okayama University Graduate School of Medicine, Dentistry and Pharmaceutical Sciences, Okayama, Japan; ^3^ Center for Innovative Clinical Medicine, Okayama University Hospital, Okayama, Japan; ^4^ Okayama Medical Innovation Center, Okayama University Graduate School of Medicine, Dentistry and Pharmaceutical Sciences, Okayama, Japan; ^5^ Department of Urology, Okamura Isshindow Hospital, Okayama, Japan

**Keywords:** renal cell carcinoma, YM155, interleukin-2, IVIS, Treg, MDSCs

## Abstract

YM155, a small molecule inhibitor of the antiapoptotic protein survivin, has been developed as a potential anti-cancer drug. We investigated a combination therapy of YM155 and interleukin-2 (IL-2) in a mouse model of renal cell carcinoma (RCC). YM155 caused cell cycle arrest and apoptosis in renal cancer (RENCA) cells. Next, luciferase-expressing RENCA cells were implanted in the left kidney and the lung of BALB/c mice to develop RCC metastatic model. In this orthotopic renal and metastatic lung tumors models, YM155 and IL-2 additively decreased tumor weight, lung metastasis, and luciferin-stained tumor images. Also, the combination significantly suppressed regulatory T cells and myeloid-derived suppressor cells compared with single agent treatment. We suggest that a combination of YM155 and IL-2 can be tested as a potential therapeutic modality in patients with RCC.

## INTRODUCTION

Renal cell carcinoma (RCC) is the most common malignant tumor of the kidney [1, 2]. Approximately 30% of patients with RCC have metastasis when diagnosed [3], and one-third develop recurrence or metastasis after initial treatment [4]. High-dose interleukin-2 (IL-2) therapy was approved in 1992 by the US Food and Drug Administration for treatment of metastatic RCC [5]. Recently, the development of therapies that target vascular endothelial growth factor (VEGF) and mammalian target of rapamycin (mTOR) pathways, including sorafenib [6], sunitinib [7], temsirolimus [8], and everolimus [9] have exhibited angiogenesis-inhibitory effects. Nevertheless, the development of resistance to targeted drugs has resulted in disease progression without durable long-term complete remission in metastatic RCC, as with IL-2 treatment, which offers the only potentially curative treatment in patients with advanced disease [10, 11]. However, the complete, durable response rates of immunotherapy have been shown to be less than 10% in select patients receiving intensive care [12, 13]. Due to its limited use and efficacy, an effective treatment strategy that includes IL-2 immunotherapy combined with other methods is required to improve the efficacy of IL-2 immunotherapy.

Survivin, encoded by the *BIRC5* gene, inhibits apoptosis and regulates mitosis [14, 15]. Survivin is undetectable or expressed at extremely low levels in normal tissue, whereas it is upregulated in many malignant tumors and plays a critical role in cancer progression and treatment resistance [16]. Survivin expression has been shown to be an independent prognostic marker of pathologic characteristics and clinical outcomes in RCC [17, 18]. These characteristics make survivin a potential therapeutic target. YM155, a novel selective small molecule survivin inhibitor, has demonstrated antitumor activities in a wide variety of mouse models of tumor xenografts [19,20] and has demonstrated synergistic antitumor activities in lung cancer, breast cancer, and leukemia when combined with other antitumor agents [21–23].

In this work, we evaluated the antitumor activities of YM155 alone and in combination with IL-2 in a mouse model of RCC. We found that YM155 treatment downregulated survivin expression in renal cancer (RENCA) cells, inhibited renal carcinoma cell proliferation, and induced cell apoptosis *in vitro*. Furthermore, the combination of YM155 and IL-2 demonstrated an additive antitumor effect on both orthotopic renal and metastatic lung tumors in a RENCA mouse model of RCC and induced the additional downregulation of peripheral myeloid-derived suppressor cells (MDSCs) and regulatory T cells (Tregs) *in vivo*.

## RESULTS

### Survivin was highly expressed in RENCA cells

**Figure 1:**
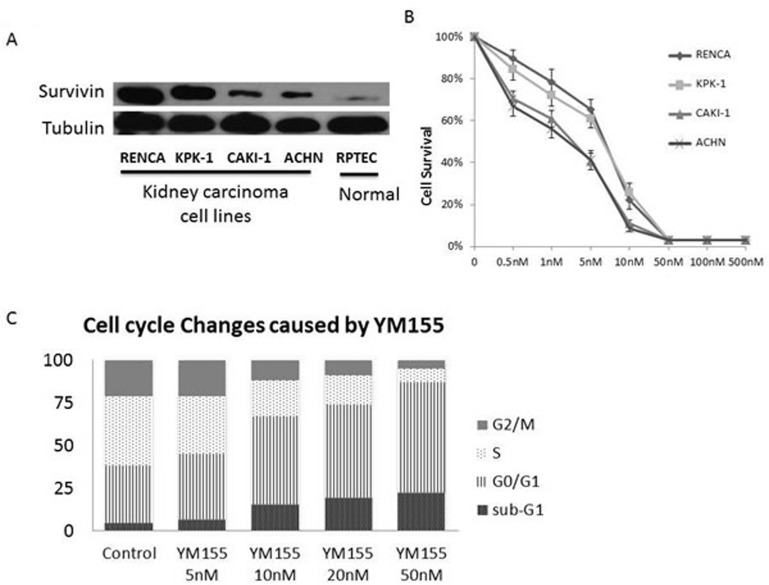
Survivin was highly expressed in renal carcinoma cell lines YM155 inhibited renal carcinoma cell proliferation and induced cell apoptosis. **A.** Western blot analysis of survivin expression levels in renal carcinoma cell lines of mice (RENCA) and humans (KPK-1, CAKI-1, ACHN), and normal cell line (RPTEC). **B.** XTT cell proliferation assay results of the renal carcinoma cell lines RENCA, KPK-1, CAKI-1, and ACHN following treatment with serial YM155 dilutions for 72 h. **C.** PI staining/FACS analyses showed increased percentages of sub-G1 and G0/G1 fractions in RENCA cells in a dose-dependent manner 24 h after YM155 treatment.

We measured survivin levels in several human and mouse renal cell lines by Western blot analysis; the highest levels were demonstrated in RENCA cells, followed by KPK-1, CAKI-1, and ACHN (Figure 1A). Survivin levels in normal cell line RPTEC were extremely low.

### YM155 inhibited renal carcinoma cell proliferation and induced cell apoptosis

The antiproliferative activity of YM155 against the renal carcinoma cell lines RENCA, KPK-1, CAKI-1, and ACHN was examined. As shown in Figure 1B, YM155 inhibited cell proliferation in a dose-dependent manner. These renal carcinoma cell lines were sensitive to YM155 treatment with 50% inhibition concentrations of approximately 3–8 nM. PI staining/flow cytometric analysis showed an increase in the percentages of sub-G1 and G0/G1 fractions in RENCA cells 24 h after YM155 treatment in a dose-dependent manner (Figure 1C).

### YM155 treatment downregulated survivin expression in RENCA cells

**Figure 2:**
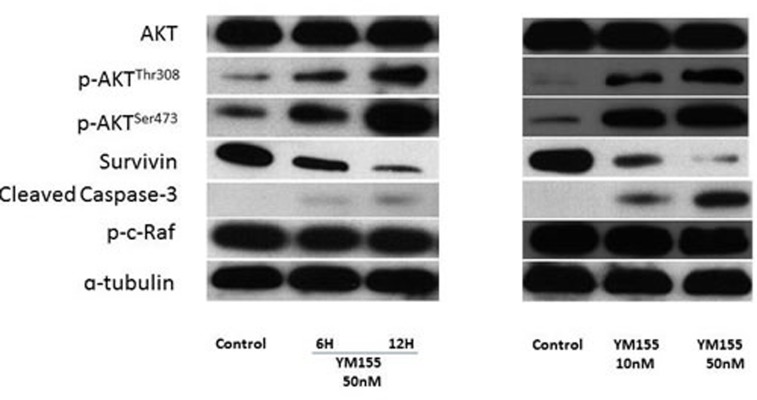
YM155 treatment downregulated survivin expression in RENCA cells **A.** After treatment with YM155 (50 nM) for 6 and 12 h, whole cell lysates were subjected to Western blot analysis against anti-survivin, AKT, phospho-Akt (Ser 473), phospho-Akt (Thr308), cleaved caspase-3, phospho-c-Raf, and tubulin. **B.** After 48 h of treatment with YM155, whole cell lysates were subjected to Western blot analysis with the indicated antibodies.

We investigated whether YM155 could inhibit survivin expression in RENCA cells, a mouse renal carcinoma cell line, and found that YM155 significantly suppressed survivin expression in a time- and dose-dependent manner (Figure 2A and 2B, respectively) and induced increasing expression of cleaved caspase-3. Interestingly, the addition of YM155 resulted in higher expression levels of phospho-Akt (Ser473) and phospho-Akt (Thr308) compared with those in the control group, whereas it did not impact expression levels of other proteins in the AKT pathway, such as AKT and phospho-c-Raf.

### Potent additive antitumor effects of the combination of YM155 and IL-2 on both orthotopic and metastatic lung tumors in a RENCA mouse model of RCC

**Figure 3:**
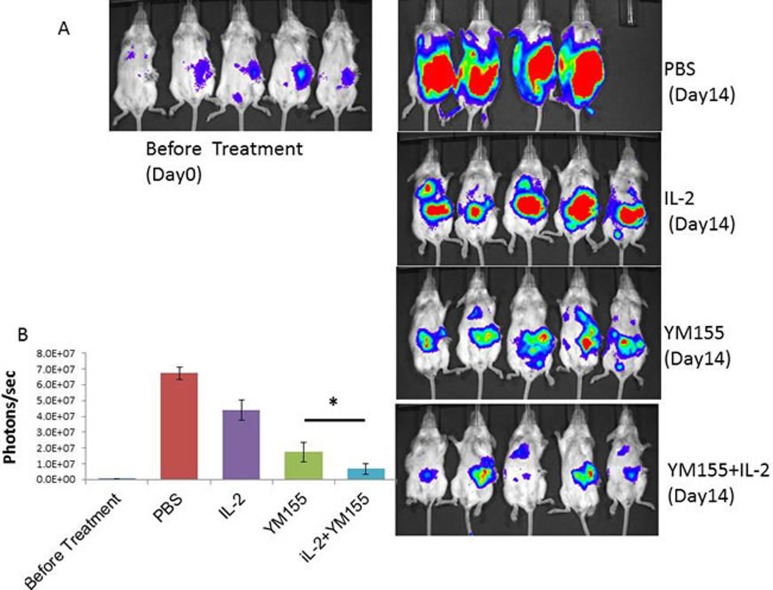
Potent additive antitumor effects following YM155 and IL-2 combination treatment on both orthotopic and metastatic lung tumors in a RENCA mouse model of RCC **A.** Bioluminescence images of mice from each treatment group are shown 14 days after treatment. One mouse in the PBS group died on post-treatment day 10. Stable luciferase-transfected RENCA cells in tumors were imaged after luciferin injection using the IVIS instrument. **B.** Tumor volume was determined by region-of-interest analysis of total photons per second. Mean values were analyzed among groups. *Significant differences were observed in comparisons of groups receiving IL-2 or YM155 alone (*p* < 0.05).

**Figure 4:**
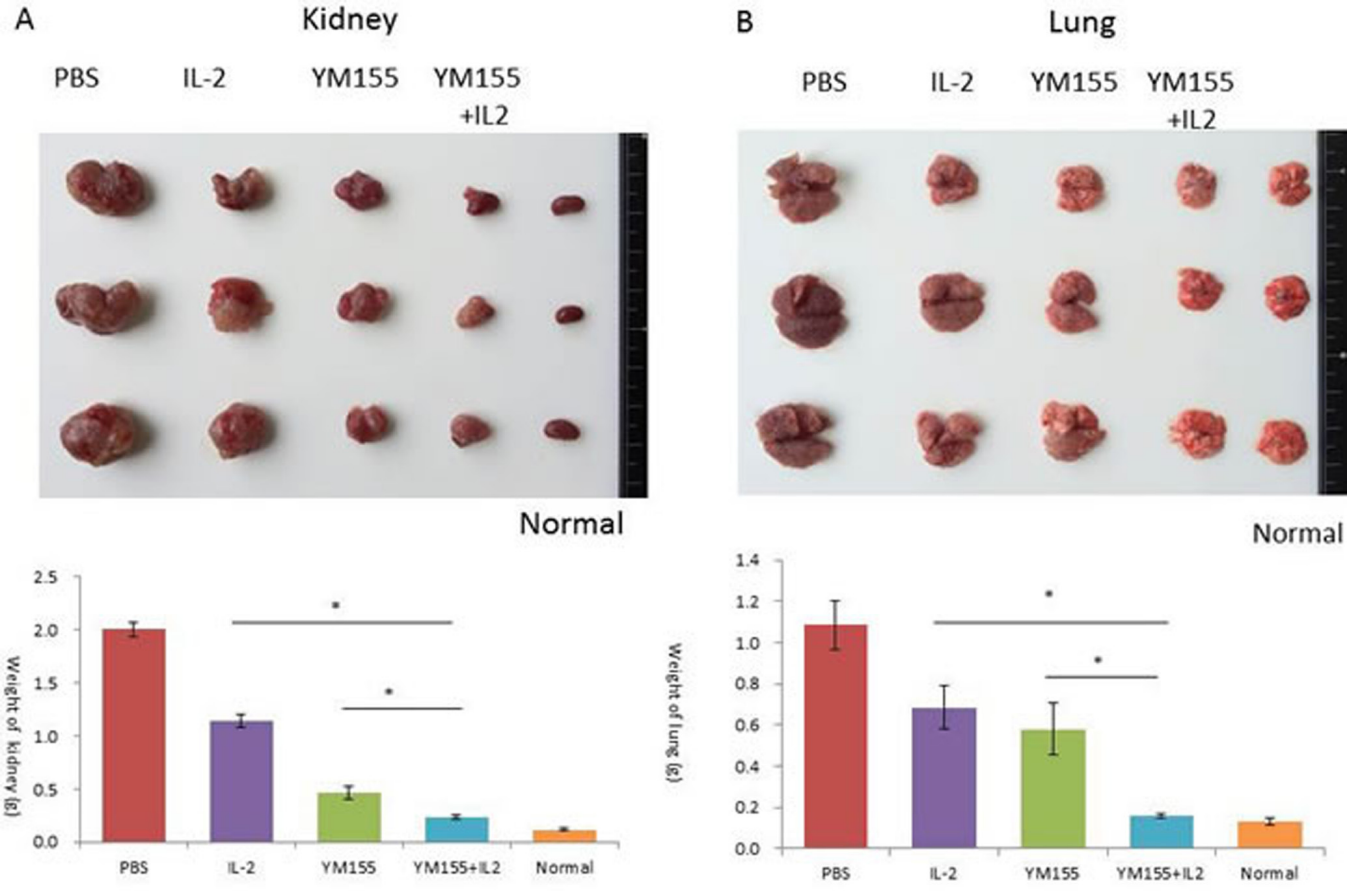
Antitumor effects of YM155 and/or IL-2 treatment on the growth of orthotopic and lung RENCA tumors in BALB/c mice **A.** A representative macroscopic view of a resected left kidney with orthotopic tumors from the indicated treatment group. The weight of the kidney was measured and mean values were analyzed among groups. **B.** A representative macroscopic view of lung tissue with metastatic tumors of the indicated treatment group is shown. The weight of the lung was measured and mean values were analyzed among groups. *A. significant difference was observed in comparison with groups receiving IL-2 or YM-155 alone.

We conducted a therapeutic trial with YM155 alone, IL-2 alone, and the combination of YM155 with IL-2 to determine their antitumor effects on the progression of both orthotopic and lung metastatic tumors in a luciferase-expressing RENCA mouse model of RCC. The therapeutic efficacy of a 1-week course of treatment with YM155 (1 mg/kg per day), a 2-week course of treatment with IL-2 with an interval of 3 days (16000 U/mouse/3 times), and the combination of YM155 and IL-2 was evaluated by demonstration of decreased tumor volume, analyzed with an*in vivo* imaging and analysis (IVIS) instrument (Figure 3A and 3B), and lower weights of orthotopic renal and metastatic lung tumor tissues (Figure 4A and 4B, respectively).

As shown in Figure 3B, tumor volume, as analyzed by the IVIS instrument, was significantly smaller following YM155 and IL-2 combination therapy, as compared with treatment with IL-2 or YM155 alone (*p* = 0.000 and 0.026, respectively). Furthermore, the tissue weights of the removed left kidney and bilateral lungs revealed that the combination of YM155 with IL-2 induced significant inhibition of tumor growth 14 days after treatment (Figure 4A and 4B, respectively). The combination of YM155 and IL-2 induced a significant antitumor effect in orthotopic renal and lung metastatic carcinomas compared with that induced by administration of either agent alone. The tissue weights of the orthotopic tumor and metastatic lung tumors were correlated to luciferase expression levels, as detected by the IVIS instrument, among the treatment groups.

### *In vivo* synergistic effects of YM155 treatment on the downregulation of peripheral MDSCs and Tregs induced by IL-2 administration

**Figure 5:**
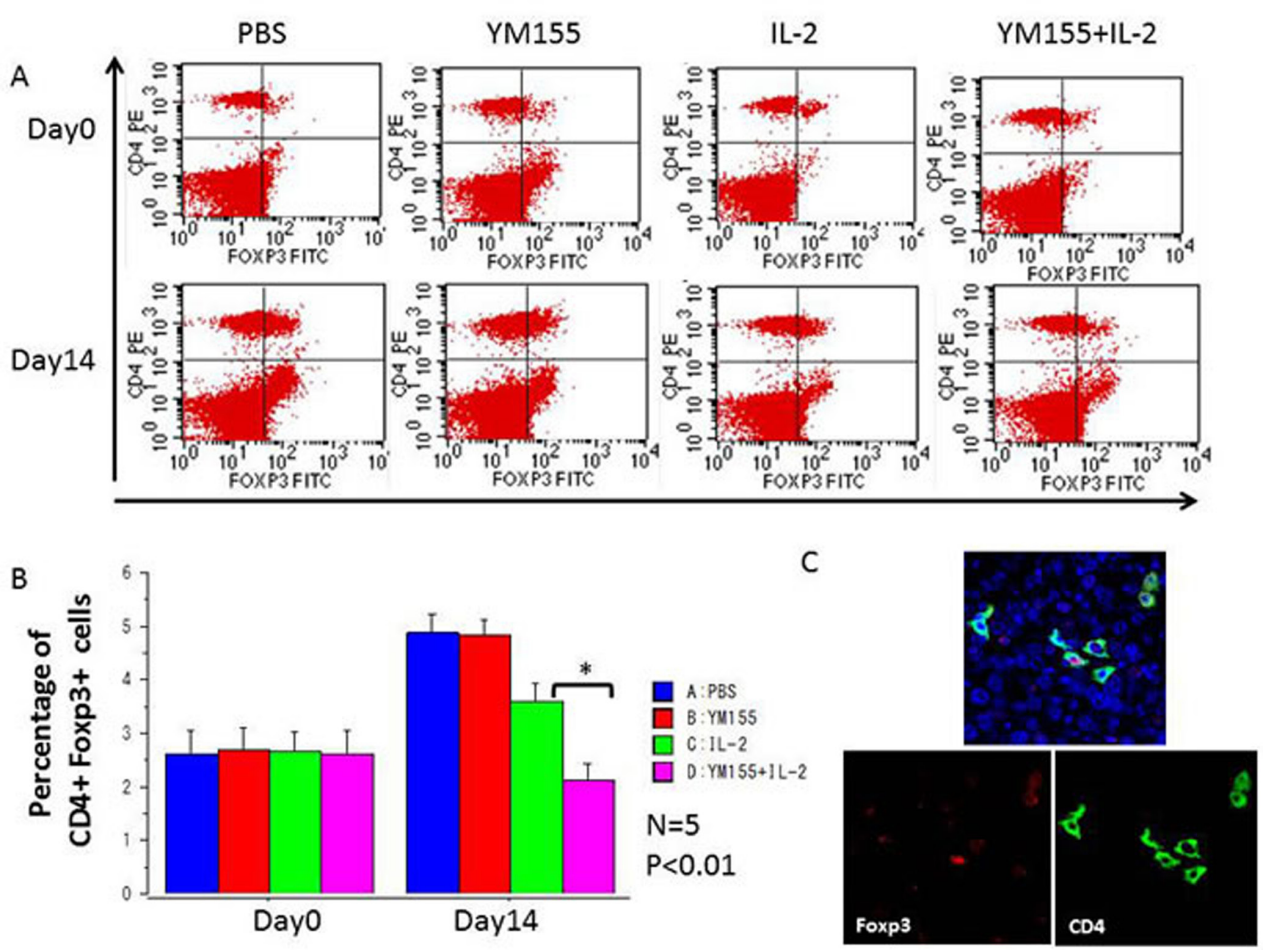
The percentages of peripheral CD4+ Foxp3^+^ nTregs among total lymphocytes in each mouse were analyzed using FACS **A.** Representative FACS data are shown. Blood samples were collected before initial treatment on day 0 and after treatment on day 14. **B.** The percentages of peripheral CD4+ Foxp3^+^ nTregs among total lymphocytes were quantified by FACS analysis and are shown for the indicated groups and times. Mean values were analyzed among groups. *A. significant difference was observed in comparison with the group receiving IL-2 alone. **C.** Three-color immunofluorescence analysis of Foxp3 (green), CD4 (red), and DAPI (blue) cells in lung tissues among groups receiving YM155 and IL-2 combination treatment.

**Figure 6:**
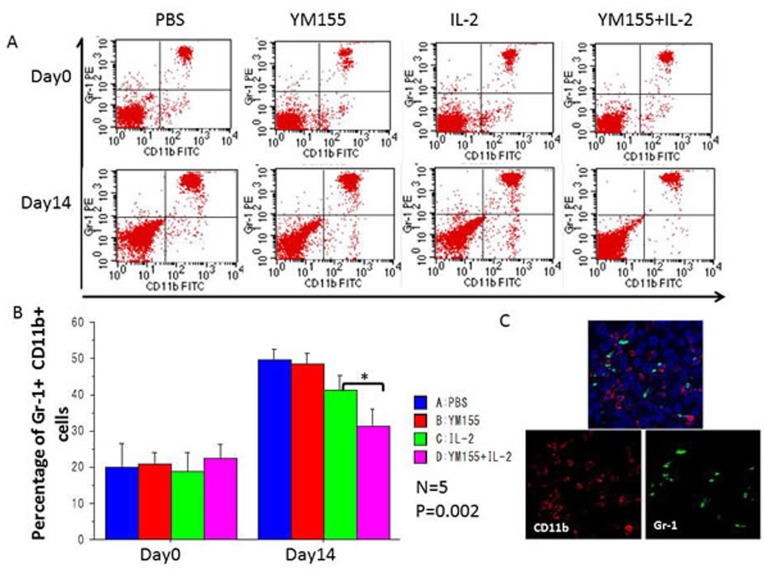
The percentages of peripheral Gr-1^+^ CD11b^+^ MDSCs among total lymphocytes in each mouse were analyzed using FACS **A.** Representative FACS data are shown. Blood samples were collected before initial treatment on day 0 and after treatment on day 14. **B.** The percentages of peripheral Gr-1^+^ CD11b^+^ MDSCs among total lymphocytes were quantified by FACS analysis and are shown for the indicated groups and times. Mean values were analyzed among groups. *A. significant difference was observed in comparison with the group receiving IL-2 alone. **C.** Three-color immunofluorescence analysis of Gr-1 (green), CD11b (red), and DAPI (blue) cells in lung tissues among treatment groups.

Populations of MDSCs and Tregs, which act as negative regulators of immune responses, are routinely elevated in patients with progressive cancer (molecular mechanisms that regulate MDSC differentiation and function). Therefore, it is increasingly clear that successful cancer immunotherapy will require limiting the immunosuppressive effects of these cell populations (coordinated myeloid cell regulation by tumors). To explore the potential mechanisms underlying the potent antitumor effects elicited by the combination of YM155 and IL-2, the percentages of Gr-1^+^ CD11b^+^ MDSCs and CD4^+^ Foxp3^+^ Tregs in peripheral blood were assessed. Before treatment, the percentages of populations of Gr-1 ^+^ CD11b^+^ MDSCs and CD4^+^ Foxp3^+^ Tregs were similar among all groups (Figures 5A and 6A, respectively), whereas the percentages of these populations in the IL-2-treated group were reduced in comparison with those in the PBS-treated group after 2 weeks of treatment. Furthermore, the population of Gr-1^+^ CD11b^+^ MDSCs (Figure 5B) and CD4^+^ Foxp3^+^ Tregs (Figure 6B) was significantly downregulated following treatment with the combination of YM155 and IL-2 compared with that following IL-2 treatment. Next, we investigated Treg and MDSC expression in orthotopic and metastatic lung tumors by immunofluorescence staining, which confirmed the presence of Gr-1^+^ CD11b^+^ MDSCs (Figure 5C) and CD4^+^ Foxp3^+^ Tregs (Figure 6C) in RCC metastatic lung tumor tissues following treatment with the combination of YM155 and IL-2. In addition, histopathological analysis of liver and other body tissue specimens indicated no histological damage in any treatment group (data not shown).

## DISCUSSION

Survivin is highly expressed in cancer and correlated with advanced disease, treatment resistance, and metastasis [16]. YM155, a novel imidazolium-based compound, has been shown to selectively suppress survivin expression in several cell lines [19, 20].

IL-2 administration is the only systemic treatment currently available that is capable of achieving a curative effect in patients with metastatic RCC. In addition, the efficacy of molecular-targeted drugs for treatment of RCC has been demonstrated, and improved therapeutic responses are expected with treatment with the combination of this class of drugs and cytokine therapy. The objective of this study was to develop an effective clinical therapy in patients with RCC to further improve efficacy [12, 13, 24].

MDSCs and Treg cells are major components of the immune suppressive tumor microenvironment [25, 26]. Both cell types proliferate systematically in preclinical tumor models and promote T-cell dysfunction, which in turn favors tumor progression. MDSCs act to suppress antitumor immunity through a number of diverse mechanisms. T-cell activation is suppressed by the production of reactive oxygen species and arginase, which induces nitration of T-cell receptors and induction of Tregs [26, 27].

In this study, the effects of the combination therapy of IL-2 and YM155 were evaluated in three animal models of RCC. The results showed that the combination treatment had a greater antitumor efficacy in a murine orthotopic renal and metastatic lung tumor model compared with the efficacy of treatment with either drug alone. Therefore, we expected synergistic antitumor effects by the combination of these agents or agents acting independently via different modes.

At first, our *in vitro* assay with mouse RCC cells demonstrated the cytotoxic effects of YM155, whereas no cytotoxic effect was induced by treatment with IL-2 alone, which was reasonable because IL-2 was not able to exhibit anticancer effects in the absence of immune cells. Previous studies showed that YM155 is an effective survivin inhibitor, and YM155 indeed induced dramatic survivin downregulation by known mechanisms of resistance to mTOR-inhibitor therapy, which included mutations in FKBP-12 [21, 22] and mTOR, PI3K/AKT [23] pathway activation, increased ERK/MAPK signaling, and activation of PIM kinases, among others [21, 28]. In our study, we analyzed molecular events related to YM155-treated RCC cells by dosage and time and found that YM155 upregulated expression levels of the PI3K/AKT pathway members p-AKT^Thr308^, p-AKT^Ser473^, and cleaved caspase-3, suggesting that YM155 may activate the AKT family to promote cell cycle arrest and apoptosis of RENCA cells.

A previous study reported that YM155 treatment sensitized myeloma cells to T cell-mediated antitumor effects and, more importantly, can overcome the microenvironment-mediated resistance of cancer cells to T cell treatment [29]. Mice with tumors in human MSC-coated scaffolds were treated with T cells alone, YM155 alone, or the combination of both. YM155 and T cell immunotherapy induced tumor regression on the evaluation of tumor growth based on bioluminescent signals. Moreover, the combination of YM155 with T cells demonstrated a substantial antitumor effect. These findings suggest that the repression of survivin with the small-molecule YM155 synergized with cytotoxic T lymphocytes and abrogated cell adhesion-mediated immune resistance (CAM-IR) both *in vitro* and *in vivo* [29]. YM155 can be used as a CAM-IR inhibitor in combination with IL-2 resistance of RCC.

In *in vivo* experiment, intravenous administration of YM155 killed cancer cells in both the orthotopic renal tumor and metastatic lung tumors, while there was no definite histological damage in the examined normal organs.

The current study revealed that the combined IP administration of YM155 plus IL-2 induced robust therapeutic effects on both orthotopic renal and metastatic lung tumors.

In mice, Treg cells occur as two distinct classes: natural Treg (nTreg) cells, which comprise 5%–10% of CD4^+^ cells, and induced Treg cells, which are the cellular components of peripheral immune tolerance. nTreg cells mature within the thymus and express the Foxp3 transcription factor. Experimental evidence indicates that nTreg cells exist without peripheral antigenic stimulation. To examine the anticancer immunomodulation in each mouse, in this study, the proportion of peripheral CD4^+^ Foxp3^+^ nTreg [24] and Gr-1^+^ CD11b^+^ MDSCs [30] were measured by FACS analysis.

Although treatment with YM155 alone had no inhibitory effect on nTreg and MDSC populations, these populations were significantly downregulated after YM155 and IL-2 combination therapy in comparison with treatment with IL-2 alone. Therefore, an *in vivo* synergistic effect of YM155 treatment on the IL-2-induced downregulation of peripheral Treg and MDSC populations was observed in the current RCC model. This immunological synergistic effect on suppression of Tregs and MDSCs may explain the robust antitumor therapeutic effects of YM155 and IL-2 combination therapy.

Several studies have investigated IL-2 as a T cell growth factor that induce Fas-mediated elimination of Tregs and MDSCs from the tumor microenvironment and elicit synergistic antitumor responses coincident with the efficient removal of Tregs and MDSCs in cancer models [31]. We previously reported that IL-2 is a T cell growth factor that is thought to play a critical role in the regulation of T cell-dependent immune responses. High-affinity IL-2 receptor-mediated cell signaling is critical to Treg regulation *in vivo* [30]. Therefore, it appears that the therapeutic effect of IL-2 is at least partially due to immunological activation by the downregulation of Tregs and MDSCs. These findings may provide a clue to clarify the mechanisms of synergistic antitumor immunological responses elicited by the combination of YM155 and IL-2. Considering the rationale for the efficacy of YM155 combined with IL-2, different independent modes of action of IL-2, which induce antitumor responses, and the action of YM155, which causes apoptosis, may act synergistically to kill RCC cells *in vivo*. In the background of lymphocyte populations, IL-2 activates suppressor T cells (Treg) in a normal state of immunosuppression. In contrast, IL-2 readily stimulates cytotoxic T cells [12, 31]. The results of the present study revealed that IL-2 stimulated a positive immune response and reduced populations of Treg cells and MDSCs when combined with YM155 against the tumor immune response.

In conclusion, the combined administration of YM155 and IL-2 exhibited a potent therapeutic effect in a murine model of RCC without any definite toxicities. These findings suggest combination therapy for both primary and metastatic RCC.

## MATERIALS AND METHODS

### Cell and cell culture

The human kidney clear cell carcinoma cell line Caki-1, adenocarcinoma cell lines ACHN and KPK-1, murine kidney carcinoma cell line RENCA, and Human Renal Proximal Tubule Cells (RPTEC) were obtained from the American Type Culture Collection (Rockville, MD, USA). All cells were maintained in Roswell Park Memorial Institute 1640 medium supplemented with 10% fetal bovine serum or Dulbecco's modified Eagle's medium (Invitrogen, Carlsbad, CA, USA) and cultured at 37°C in an atmosphere of 5% CO_2_.

### Animals

Female 8-week-old BALB/c mice were purchased from Japan SLC, Inc. (Hamamatsu, Japan) and housed in a specific pathogen-free environment with free access to food and water at the laboratory animal center of Okayama University (Okayama, Japan). Mice were acclimated to this environment for more than 1 week before beginning the experiments. Animals were housed and handled in accordance with the guidelines of the Okayama University Animal Research Committee.

### Drugs and antibodies

The survivin suppressor YM155 was obtained from Selleck Chemicals Inc. (Houston, TX, USA). IL-2 was kindly provided by Shionogi Co. (Osaka, Japan). Primary antibodies against survivin, phosho-PDK1, phosho-PTEN, AKT, phosho-AKT (Ser473), phosho-AKT (Ser308), phosho-GSK-3β (Ser9), cleaved caspase-3, phosho-c-Raf, and ɑ-tubulin were purchased from Cell Signaling Technology, Inc. (Danvers, MA, USA).

### XTT proliferation assay

The effect of YM155 on cell growth was determined using a cell proliferation kit that employs the sodium 3′-[1-(phenylaminocarbonyl)-3,4-tetrazolium]-bis (4-methoxy-6-nitro) benzene sulfonic acid hydrate (XTT) assay (Roche Diagnostics, Indianapolis, IN, USA). Aliquots of 1 × 10^3^ RENCA cells were seeded in 96-well plates and incubated with medium alone or serial dilutions of YM155. After 72 h, an XTT labeling mixture containing an XTT labeling reagent and an electron coupling reagent was added to the culture. After a 4-h incubation, the absorbance at 450–500 nm with a reference wavelength at 650 nm was recorded using a microplate reader (model 680; Bio-Rad Laboratories, Inc., Hercules, CA, USA).

### Flow cytometry

Treated cells were washed with PBS, fixed in 75% ethanol for 2 h at 4°C, and then suspended and incubated in sodium citrate solution containing RNase A (Roche Diagnostics) and propidium iodide (PI) (Life Technologies, Grand Island, NY, USA) in the dark for 30 min at 4°C. Flow cytometric analyses were performed using a BD FASC Cabibur cytometer (BD Biosciences, San Jose, CA, USA) and CellQuest software (BD Biosciences). Blood samples from mice were collected into tubes containing ethylenediaminetetraacetic acid (EDTA). The samples were then incubated with phycoerythrin-labeled anti-mouse CD4 antibody and fluorescein isothiocyanate-labeled anti-mouse Foxp3 antibody or phycoerythrin-labeled anti-mouse Gr-1 antibody and fluorescein isothiocyanate-labeled anti-mouse CD11b antibody (all, eBioscience, Inc., San Diego, CA, USA) for 1 h at 4°C and washed twice with PBS. Next, the samples were resuspended in 250 μL of cold PBS and analyzed using a fluorescence-activated cell sorter (FACS) (Calibur flow cytometer; BD Biosciences) with gating.

### Western blot analysis

Cells were lysed with lysis buffer that contained 50 mM HEPES (4-(2-hydroxyethyl)-1-piperazineethanesulfonic acid, pH 7.4), 250 mM NaCl, 1 mM EDTA, 1% NP-40, 1 mM dithiothreitol, 1 mM phenylmethylsulfonyl fluoride, 5 μg/mL of leupeptin, 5 μg/mL of aprotinin, 2 mM Na_3_VO_4_, 1 mM NaF, and 10 mM Δ-glycerophosphate. Approximately 100 μg of protein was suspended in Laemmli sample buffer (BD Biosciences), boiled, and separated by sodium dodecyl sulfate-polyacrylamide gel electrophoresis. The gels were electrotransferred to a polyvinylidene difluoride membrane (Bio-Rad Laboratories, Inc.), which was then incubated with the indicated primary antibodies in 5% BSA or nonfat milk in TBS-T overnight at 4°C. Next, the membranes were washed and immediately incubated with anti-rabbit or anti-mouse horseradish peroxidase-conjugated secondary antibodies. Specific proteins were detected by exposing membranes to x-ray film after incubation with an enhanced chemiluminescence reagent (ECL kit; Amersham Pharmacia Biotech, Chandler, AZ, USA).

### Imaging of RCC tumors in live mice

Tumors derived from stable luciferase-transfected RENCA cells were imaged to observe luciferase expression. Briefly, the animals were anesthetized and then injected IP with luciferin (a substrate of luciferase) at 150 mg/kg in a volume of 100 μL. Images were captured at a peak time of 20 min after injection using an IVIS-200 Imaging System (Xenogen Corporation, Alameda, CA, USA) and then processed using Living Image software (Xenogen Corporation) by region-of-interest analysis of the total photons per second for each tumor with appropriate background subtraction.

### Therapeutic studies

Luciferase-expressing RENCA cells were implanted to the subrenal capsule of the left kidney and tail vein, respectively, to produce a mouse RCC model with an orthotopic tumor and metastatic lung tumors. The mice were randomly divided into four groups with an even distribution of IVIS values. Group 1 received intraperitoneal (IP) injection of 100 μL of phosphate-buffered saline (PBS) as a vehicle control; group 2 received YM155 alone (1 mg/kg body weight per day for 1 week by IP injection); group 3 received IL-2 alone (6000 U of recombinant IL-2 by IP injection on days 0, 4, and 8 of treatment); and group 4, the combination therapy group, received YM155 and IL-2 (dose and dosing schedule the same as in group 2 plus group 3). Tumor imaging was performed and tumor volume was analyzed in all groups on day 14 post-treatment. The mice were sacrificed, and the weights of the orthotopic tumor in the left kidney and sections of bilateral lung tissues with metastatic growth were measured.

### Immunofluorescence staining

After deparaffinization and rehydration of tissue sections, antigens were unmasked by blocking with 5% normal goat serum and 0.3% Triton™ X-100 in PBS and overnight incubation with diluted primary antibodies (purified anti-mouse Ly-6G/Ly-6C, BioLegend, San Diego, CA, USA; CD11b, Life Technologies; CD4, Santa Cruz Biotechnology, Inc., Dallas, TX, USA; Foxp3, Novus Biologicals LLC, Littleton, Co, USA) in a humidified chamber at 4°C. All specimens were then washed with PBS and immediately incubated with fluorochrome-conjugated secondary antibody [Alexa Fluor 488 goat anti-rat IgG, Rhodamine Red™-X Goat Anti-Rabbit IgG (H + L), Life Technologies] diluted in antibody dilution buffer for 1 h at room temperature in the dark. Slides were covered with Mounting Medium with DAPI (Vector Laboratories, Inc., Burlingame, CA, USA).

### Statistical analysis

Data are expressed as mean values ± standard deviation (SD). One-way analysis of variance followed by Bonferroni's post-hoc comparison tests was performed for comparisons among multiple groups. A *p* value < 0.05 was considered statistically significant.
